# Supplementary material for: Isolation and Characterization of a ssDNA Aptamer against Major Soluble Antigen of Renibacterium salmoninarum
Source: Molecules. 2022 Mar 12;27(6):1853. doi: 10.3390/molecules27061853 (PMC8951219; doi:10.3390/molecules27061853)
Supplement: Supplementary file 1 [file molecules-27-01853-s001.zip › molecules-1569739-supplementary.pdf]

Supplementary material

Isolation and Characterization of a ssDNA Aptamer against Major Soluble Antigen of *Renibacterium salmoninarum*

Brady Layman [1], Brian Mandella [1], Jessica Carter [1], Haley Breen [1], John Rinehart [2] and Anna Cavinato [1,*]

[1] Department of Chemistry and Biochemistry, Eastern Oregon University, La Grande, OR 97850, USA; blayman@eou.edu (B.L.); bmandella@eou.edu (B.M.); jcarter@eou.edu (J.C.); hbreen@eou.edu (H.B.)
[2] Department of Biology, Eastern Oregon University, La Grande, OR 97850, USA; jrinehar@eou.edu
* Correspondence: acavinat@eou.edu

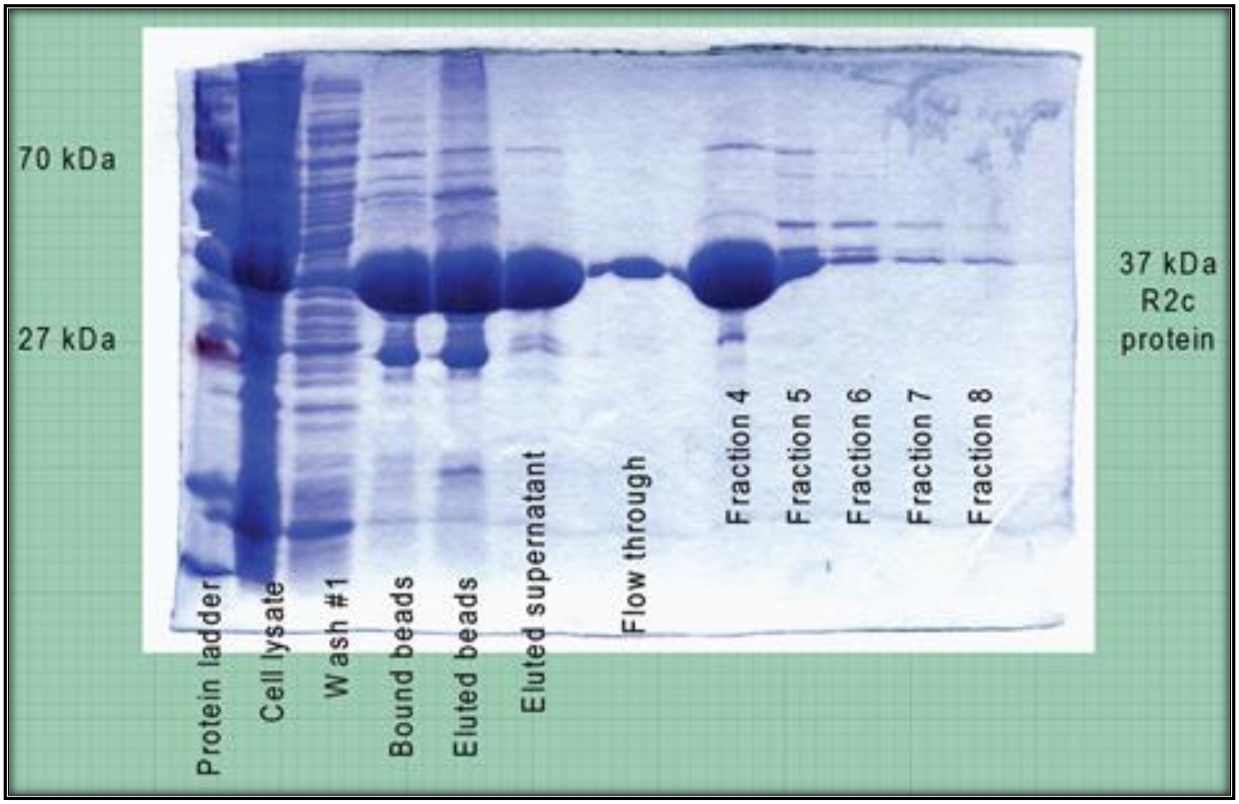

Figure S1. SDS-PAGE for the protein domain R2c. The protein was identified at 37 kDa.

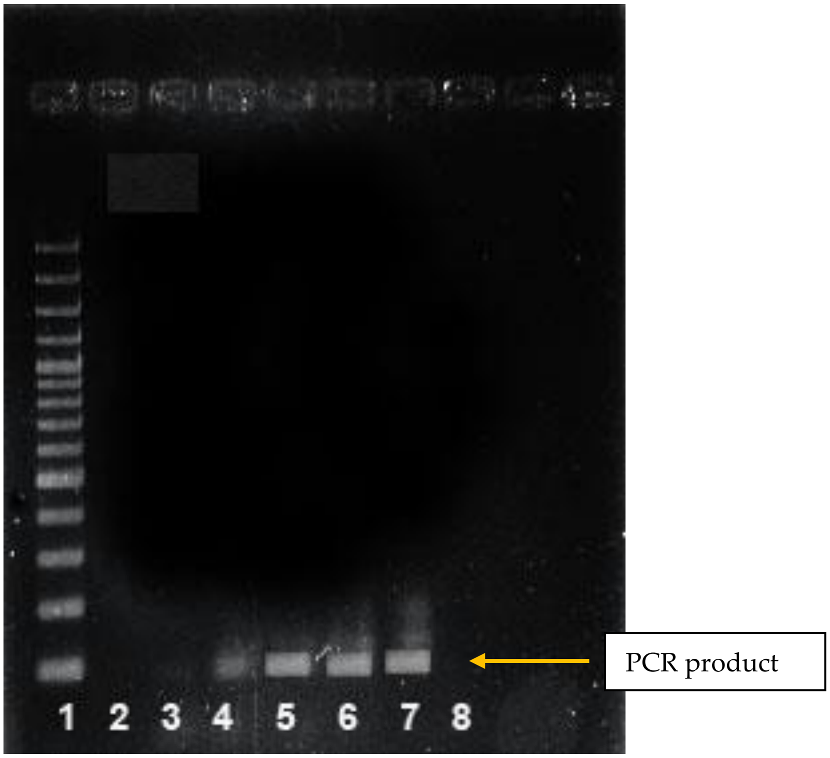

Figure S2. Optimization of aptamer pools after each GO-SELEX cycle. PCR amplification was performed in increments of 2 PCR cycles until the band of proper size was visualized on a 2.5% agarose gel by electrophoresis. In this example 10 PCR cycles represent the optimal choice with a distinct single band. Lane 1: 100 bp ladder; Lane 2: empty; Lane 3: 6; Lane 4: 8; Lane 5:10; Lane 6: 12; Lane 7: 14 PCR cycles; Lane 8: negative control.

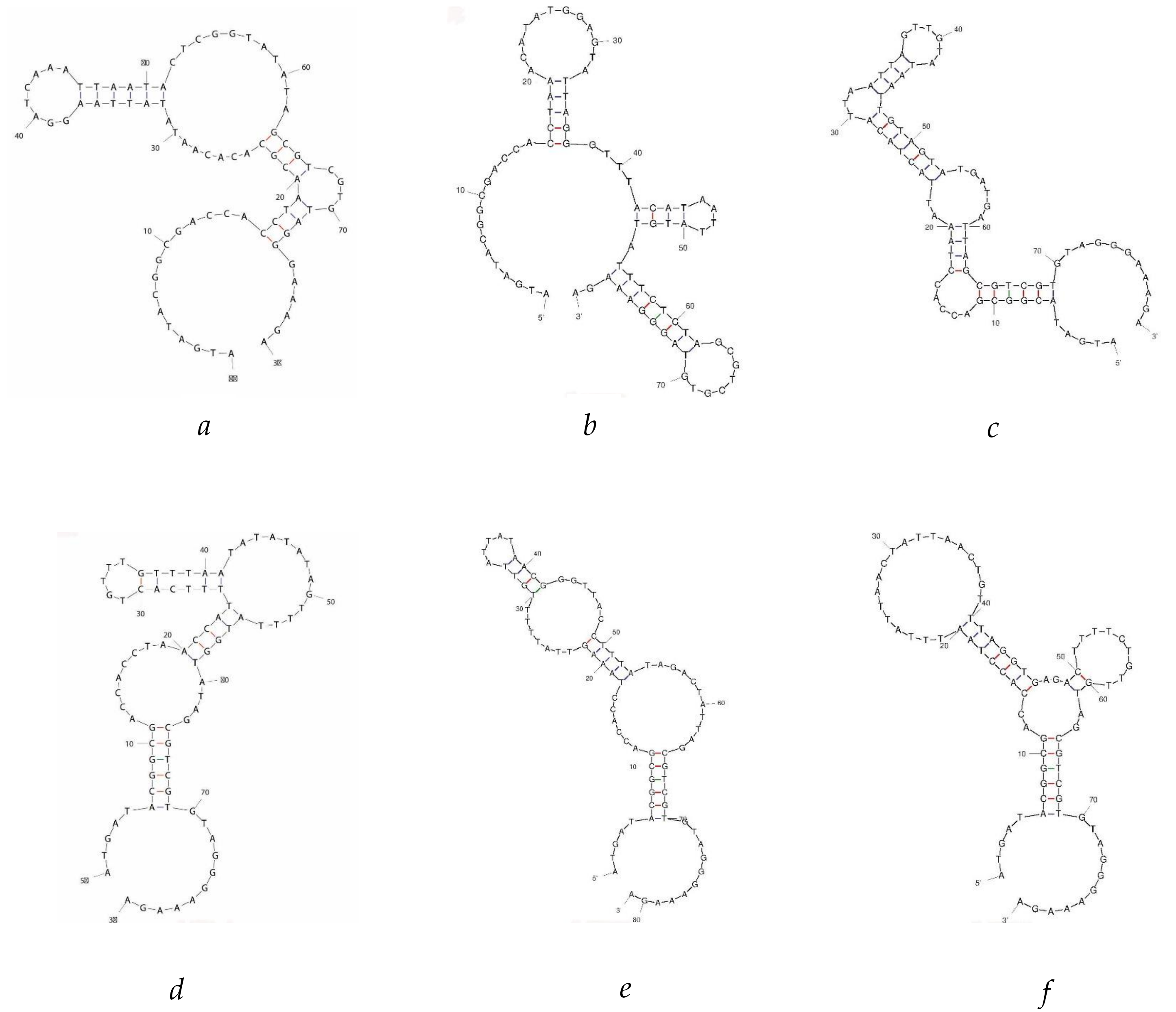

Figure S3. Minimum free energy structures predicted by mfold software at 37°C (binding buffer conditions were similar to the ones used in this study) for Aptamer-1 (a) Aptamer-2 (b) Aptamer-3 (c) Aptamer-4 (d) Aptamer-5 (e) Aptamer-6. Free energies predicted by the software for these structures from a-f are, -6.47kcal/mol, -5.797kcal/mol, -5.58kcal/mol, -7.23kcal/mol, -2.27 kcal/mole, -6.31kcal/mol, respectively.

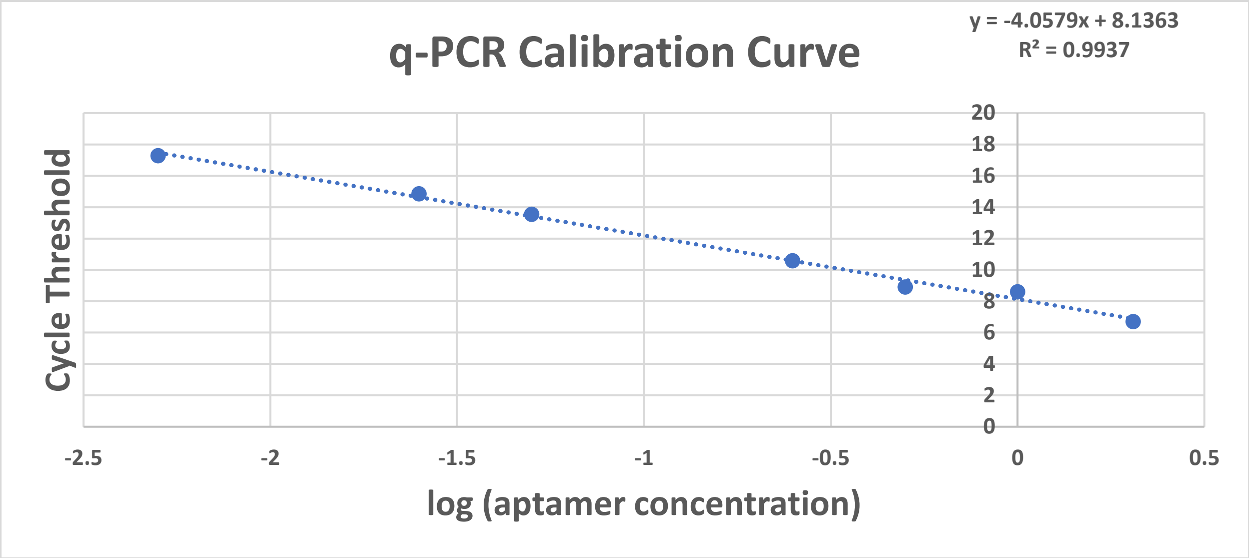

Figure S4. qPCR calibration curve showing a linear correlation between the cycle threshold and aptamer concentration in the 0.005 to 2.4 nM range.
